# Unexpected Role for IL-17 in Protective Immunity against Hypervirulent *Mycobacterium tuberculosis* HN878 Infection

**DOI:** 10.1371/journal.ppat.1004099

**Published:** 2014-05-15

**Authors:** Radha Gopal, Leticia Monin, Samantha Slight, Uzodinma Uche, Emmeline Blanchard, Beth A. Fallert Junecko, Rosalio Ramos-Payan, Christina L. Stallings, Todd A. Reinhart, Jay K. Kolls, Deepak Kaushal, Uma Nagarajan, Javier Rangel-Moreno, Shabaana A. Khader

**Affiliations:** 1 Department of Pediatrics, Division of Infectious Diseases, University of Pittsburgh School of Medicine, Pittsburgh, Pennsylvania, United States of America; 2 Department of Infectious Diseases and Microbiology, University of Pittsburgh, Pittsburgh, Pennsylvania, United States of America; 3 Faculty of Biological and Chemical Sciences, Autonomous University of Sinaloa, Culiacan, Sinaloa, Mexico; 4 Department of Medicine, Division of Allergy, Immunology and Rheumatology, University of Rochester Medical Center, Rochester, New York, United States of America; 5 Department of Molecular Microbiology, Washington University in St. Louis, St. Louis, Missouri, United States of America; 6 Richard King Mellon Foundation Institute for Pediatric Research, Children's Hospital of Pittsburgh of University of Pittsburgh Medical Center, Pittsburgh, Pennsylvania, United States of America; 7 Tulane National Primate Center, New Orleans, Louisiana, United States of America; 8 Department of Pediatrics, University of North Carolina, Chapel Hill, Chapel Hill, North Carolina, United States of America; Portland VA Medical Center, Oregon Health and Science University, United States of America

## Abstract

*Mycobacterium tuberculosis (Mtb)*, the causative agent of tuberculosis (TB), infects one third of the world's population. Among these infections, clinical isolates belonging to the W-Beijing appear to be emerging, representing about 50% of *Mtb* isolates in East Asia, and about 13% of all *Mtb* isolates worldwide. In animal models, infection with W-Beijing strain, *Mtb* HN878, is considered “hypervirulent” as it results in increased mortality and causes exacerbated immunopathology in infected animals. We had previously shown the Interleukin (IL) -17 pathway is dispensable for primary immunity against infection with the lab adapted *Mtb* H37Rv strain. However, it is not known whether IL-17 has any role to play in protective immunity against infection with clinical *Mtb* isolates. We report here that lab adapted *Mtb* strains, such as H37Rv, or less virulent *Mtb* clinical isolates, such as *Mtb* CDC1551, do not require IL-17 for protective immunity against infection while infection with *Mtb* HN878 requires IL-17 for early protective immunity. Unexpectedly, *Mtb* HN878 induces robust production of IL-1β through a TLR-2-dependent mechanism, which supports potent IL-17 responses. We also show that the role for IL-17 in mediating protective immunity against *Mtb* HN878 is through IL-17 Receptor signaling in non-hematopoietic cells, mediating the induction of the chemokine, CXCL-13, which is required for localization of T cells within lung lymphoid follicles. Correct T cell localization within lymphoid follicles in the lung is required for maximal macrophage activation and *Mtb* control. Since IL-17 has a critical role in vaccine-induced immunity against TB, our results have far reaching implications for the design of vaccines and therapies to prevent and treat emerging *Mtb* strains. In addition, our data changes the existing paradigm that IL-17 is dispensable for primary immunity against *Mtb* infection, and instead suggests a differential role for IL-17 in early protective immunity against emerging *Mtb* strains.

## Author Summary

*Mycobacterium tuberculosis (Mtb)*, the causative agent of tuberculosis (TB), infects one third of the world's population. Among these infections, clinical isolates belonging to the W-Beijing are emerging, representing about 50% of *Mtb* isolates in East Asia, and about 13% of all *Mtb* isolates worldwide. In animal models, infection with W-Beijing strain, *Mtb* HN878, is considered “hypervirulent” resulting in increased mortality. The proinflammatory cytokine Interleukin (IL)-17 is thought to be dispensable for primary immunity against *Mtb* infection. We report here that while IL-17 is dispensable for protection against infection with lab adapted *Mtb* strains such as H37Rv, or less virulent *Mtb* clinical isolates such as *Mtb* CDC1551, IL-17 is required for early protective immunity against *Mtb* HN878 infection. The dependence on IL-17 to drive protective immunity against *Mtb* HN878 is due to the differential ability to induce high levels of IL-1β through a TLR-2-dependent mechanism, driving potent IL-17 responses, induction of the chemokine CXCL-13 and localization of T cells within lung lymphoid follicles for maximal macrophage activation and *Mtb* control. Together, our data change the existing paradigm that IL-17 is dispensable for primary immunity against *Mtb* infection, and suggests a differential requirement for IL-17 in protective immunity against some emerging *Mtb* strains.

## Introduction

*Mycobacterium tuberculosis (Mtb), the* causative agent of tuberculosis (TB), infects one third of the world's population. While most infected individuals develop latent TB, 5–10% of infected individuals develop active TB. In addition, although most infected people with latent TB remain asymptomatic, they have ∼10% lifetime risk of developing into active TB. Among these infections, clinical isolates being typed as belonging to the W-Beijing strain appear to be increasingly prevalent. In fact, recent reports show that W-Beijing family *Mtb* strains represent about 50% of *Mtb* isolates in East Asia, and are believed to account for at least 13% of all *Mtb* isolates worldwide [1]–[4]. More importantly, multiple studies have identified that W-Beijing strains are over-represented among drug resistant isolates [5], [6], and are significantly associated with human immunodeficiency virus (HIV) infection in humans [7]. In animal models, infection with *Mtb* HN878 isolate, the best studied of the W-Beijing isolates, is thought to be “hypervirulent” as it results in increased mortality and causes severe immunopathology in infected animals [8], [9]. In addition, studies suggest that *M.bovis* Bacille Calmette-Guerin (BCG) vaccination may be less protective against W-Beijing genotype *Mtb* strains [4], thus contributing to its successful recent worldwide emergence.

The immune responses that mediate protective immunity against *Mtb* infection are through the production of proinflammatory cytokines such as Interferon gamma (IFN-γ) and Tumor necrosis factor alpha (TNF-α), both cytokines that activate macrophages to mediate *Mtb* control. This is consistent with the finding that *Mtb* HN878 infection in mice induces a Type I Interferon response, which limits the generation of T helper type 1 cells (Th1), that produce IFN-γ and TNF-α [9], [10]. In addition, *Mtb* HN878 infection also inhibits the production of TNF-α in macrophages [11], suggesting that the increased virulence of *Mtb* HN878 infection may be due to the reduced generation of Th1 responses and impaired macrophage activation in the host. Interleukin-17 (IL-17) is a pro-inflammatory cytokine, well described for its role in host defense against extracellular bacterial pathogens [12]. We had previously shown that the IL-17 pathway is not required for primary immunity against infection with the lab adapted strain, *Mtb* H37Rv [13]–[15]. However, it is not known whether IL-17 has any role to play in protective immunity against infection with clinical *Mtb* isolates. In the current study, we tested whether IL-17 is required for protective immunity following infection with the hypervirulent *Mtb* HN878, and the less virulent *Mtb* CDC1551 clinical isolates. Surprisingly, we found that while lab adapted *Mtb* isolates such as *Mtb* H37Rv, or less virulent *Mtb* clinical isolates such as *Mtb* CDC1551, did not require IL-17 for early protective immunity against infection, infection with *Mtb* HN878 required the production of IL-17 for protective immunity. Our data suggest that the dependence on IL-17 to drive protective immunity against *Mtb* HN878 is due to the differential ability of *Mtb* HN878 to induce high levels of IL-1β through a TLR-2-dependent mechanism, resulting in high IL-17 production. In addition, our data show that the role for IL-17 in mediating early protective immunity against *Mtb* HN878 is through IL-17 receptor (IL-17R) signaling in non-hematopoietic cells, to induce expression of the chemokine, CXCL-13. CXCL-13 expression attracts cytokine-producing CXCR5^+^ T cells which localize near *Mtb*-infected macrophages, to form lung lymphoid follicles for optimal macrophage activation and *Mtb* control. Our novel results suggest that the protective immune requirements for emerging hypervirulent *Mtb* isolates are likely different from the requirements for lab adapted and less virulent *Mtb* isolates, and thus need to be studied independently as demonstrated here. As recent work has demonstrated a critical role for IL-17 in vaccine induced immunity against TB [16], [17], our results have far reaching implications for the design of vaccines and therapies to prevent and treat emerging *Mtb* strains such as W-Beijing strains.

## Materials and Methods

### *Mtb* infection in mice

B6 (B6), IL-17*^−/−^* [18], IL17RA^−/−^ (IL-17R^−/−^) [19], CXCR5^−/−^ [20], IL-17GFP reporter, IL-1R^−/−^, TLR2^−/−^ and ESAT-6 T-Cell Receptor (TCR) Transgenic (Tg) mice [21] were bred at the Children's Hospital of Pittsburgh or purchased from Jackson lab. ESAT-6 TCR Tg mice were also crossed to the CXCR5^−/−^ mice to generate Tg mice which were deficient in CXCR5. Experimental mice were age- and sex-matched and used between the ages of 6 to 8 wks. *Mtb* strains H37Rv, CDC1551 or HN878 were cultured in Proskauer Beck medium containing 0.05% Tween 80 to mid-log phase and frozen in 1 ml aliquots at −70°C. Mice were aerosol infected with ∼100 CFU of bacteria using a Glas-Col airborne infection system [16]. At given time points, organs were harvested, homogenized and serial dilutions of tissue homogenates plated on 7H11 plates and CFU determined. In some experiments, adenovirus over-expressing IL-17 or control adenovirus expressing luciferase vector was delivered once (5×10^8^ pfu) intratracheally.

### Ethics statement

All mice were used in accordance following the National Institutes of Health guidelines for housing and care of laboratory animals and in accordance with University of Pittsburgh Institutional Animal Care and Use Committee guidelines and were approved under Protocol 0807913. All efforts were made to minimize suffering and pain as described in this approved protocol.

### Lung cell preparation and flow cytometry

Lung cell suspensions were prepared as described [16] and single cell suspensions were stained with appropriate fluorochrome-labeled specific antibodies or isotype control antibodies. Cells were collected using a Becton Dickinson FACS Aria flow cytometer using FACS Diva software. Cells were gated based on their forward by side scatter characteristics and the frequency of specific cell types was calculated using FlowJo (Tree Star Inc, CA).

### Detection of IFN-γ and IL-17 producing cells by ELISpot assay

ESAT-6_1–20_-specific IFN-γ and IL-17-producing IA^b^-restricted T cells from infected lungs or spleen were enumerated using peptide-driven ELISpot as described [22]. Briefly, 96 well ELISpot plates were coated with monoclonal anti-mouse IFN-γ or IL-17, blocked with media containing 10% FBS. Cells from lungs and spleen were seeded at an initial concentration of 5×10^5^ cells/well and subsequently diluted two fold. Irradiated B6 splenocytes were used as APCs at a concentration of 1×10^6^ cells/well in the presence of ESAT-6_1–20_ (10 µg/ml) peptide and IL-2 (10 U/ml). After 24 hrs, plates were washed and probed with biotinylated anti-mouse IFN-γ or IL-17. Spots were visualized and enumerated using a CTL-Immunospot S5 MicroAnalyzer. No spots were detected in cultures lacking antigen or when using cells from uninfected mice.

### Bone marrow-derived macrophages and dendritic cell preparation

BMDMs and BMDCs were generated from the bone marrow of B6 or gene deficient mice. Cells were extracted from femurs and 1×10^7^ cells were plated with 10 ml of cDMEM supplemented with 20 ng/mL mouse recombinant GM-CSF (Peprotech). Cells were cultured for 3 days at 37°C in 5% CO2, after which an additional 10 ml of cDMEM containing 20 ng/ml rmGM-CSF was added. On day 7, non-adherent cells were collected by centrifugation and counted as DCs, while adherent cells were collected and used as BMDMs. Lung or BMDCs were treated with *Mtb* HN878 or *Mtb* H37Rv components, such as whole cell lysate, cell wall or lipids preparations (20 µg/ml each, BEI Resources, obtained under National Institutes of Health [NIH] contract AI-75320)

### Lung dendritic cell purification

Single cell suspensions from DNAse/collagenase-treated lung tissue were prepared as previously described. CD11c^+^ cells were isolated using magnetic anti-CD11c beads (Miltenyi Biotec Inc.), according to the manufacturer's instructions.

### In vitro *Mtb* infection

BMDCs and lung dendritic cells prepared as previously described were plated at a density of 1×10^6^ cells/ml and rested overnight. Cells were subsequently infected with different *Mtb* strains at a multiplicity of infection (MOI) of 5 for BMDCs or 0.1 for lung dendritic cells in antibiotic-free DMEM, for 24 hrs, following which total lung cell suspensions from B6 or gene deficient mice were added in a 1∶1 ratio to the infected dendritic cells, and co-cultured for 6 days.

### Generation of ESAT-6- Tg Th17 cells

Naive CD4^+^ T cells were isolated from single cell suspensions generated from lymph nodes and spleens of ESAT-6 TCR Tg mice using a positive CD4 T cell isolation kit (Miltenyi Biotech) as described [23]. For generation of Th17 cells, cells were cultured in Complete Iscove's medium containing TGFβ (5 ng/ml), IL-6 (30 ng/ml), IL-23 (50 ng/ml), anti-IL-4 (10 µg/ml), anti-IFN-γ (10 µg/ml), and IL-2 (10 U/ml) and APCs [24]. T cells were incubated for six days at 37°C and 5% CO_2_ and supplemented with an equivalent volume of media containing IL-2 (10 U/ml) on day 3. Cells were harvested on day 6 and washed twice with PBS. For adoptive transfer, 3–5×10^6^ ESAT-6 TCR Tg Th17 cells were transferred intravenously into recipient mice, following which mice were rested for 24 hours and challenged with *Mtb* H37Rv by the aerosol route.

### Generation of bone marrow chimeric mice

To generate chimeric mice, mice were given a medicated Sulfa-Trim diet containing 1.2% sulfamethoxazole and 0.2% trimethoprim (TestDiet) two weeks prior to irradiation. Mice were sub-lethally irradiated with 1000 rads in two doses (X-Rad 320). Mice were subsequently reconstituted with 10×10^6^ bone marrow cells from B6 or gene deficient mice via i.v. injection. Mice were allowed to reconstitute for 45 days while continuing to receive a Sulfa-Trim and acidified water diet following which they were used in experimental procedures.

### Immunohistochemistry

Lung lobes were instilled with 10% neutral buffered formalin and embedded in paraffin. Lung sections were stained with hematoxylin and eosin (H&E) and inflammatory features were evaluated by light microscopy (Research Histology Core, University of Pittsburgh). For immunofluorescent staining, formalin-fixed lung sections were cut, immersed in xylene to remove paraffin and then hydrated in 96% alcohol and PBS. Antigens were unmasked with a DakoCytomation Target Retrieval Solution and non-specific binding was blocked with 5% (v/v) normal donkey serum and Fc block (BD Pharmingen, San Diego, CA). Endogenous biotin (Sigma Aldrich) was neutralized by adding first avidin, followed by incubation with biotin. Sections were probed with anti-B220 to detect B cells (Clone RA3-6B2, BD Pharmingen, San Diego, CA), anti-CD3 to detect T cells (Clone M-20, Santa Cruz Biotechnology, Santa Cruz, CA), anti-CXCL13 (Clone143614, R & D Biosystems) to detect CXCL-13 expression, and inducible NO synthase (goat anti-mouse, M-19; Santa Cruz Biotechnology) and F4/80 (MCA497GA, Serotec) to detect activated macrophages within inflammatory lesions. B cell lymphoid follicles were outlined with the automated tool of the Zeiss Axioplan 2 microscope (Carl Zeiss) and total area and average size was calculated in squared microns. F4/80 macrophages expressing iNOS in five random 20× fields were enumerated per lung (n = 5 lungs) and the average was calculated. 3–5 granulomas per lobe in each group were randomly chosen to quantify CXCL13 mRNA expression in 200× fields as described before [17]. Samples were analyzed in a blinded fashion.

### In situ hybridization

Paraffin embedded tissue sections were deparaffinized and washed in ethanol. Stringent in situ hybridization (50°C with 0.1M DTT in the hybridization mix) was performed with 35S-labeled riboprobes as previously described [20]. Tissue sections were coated with NTB emulsion (Carestream/Kodak), exposed at 10°C for 14 days, counterstained with hematoxylin (Vector Laboratories) and mounted with Permount (Fisher). Images were visualized using an Olympus BX41 microscope (Olympus) and captured using a SPOT RT3 digital camera (Diagnostics Instruments).

### Protein estimation by ELISA

Protein levels for cytokines and chemokines in culture supernatants, serum or lung homogenates were measured using a mouse Luminex assay (Linco**/**Millipore).

### Statistical analysis

Differences between the means of groups were analyzed using the two tailed Student's *t*-test in GraphPad Prism 5 (La Jolla, CA).

## Results

### IL-17 is dispensable for early protective immunity against lab strain *Mtb* H37Rv, but required for protective immunity against the hypervirulent clinical isolate, *Mtb* HN878

**Figure 1:**
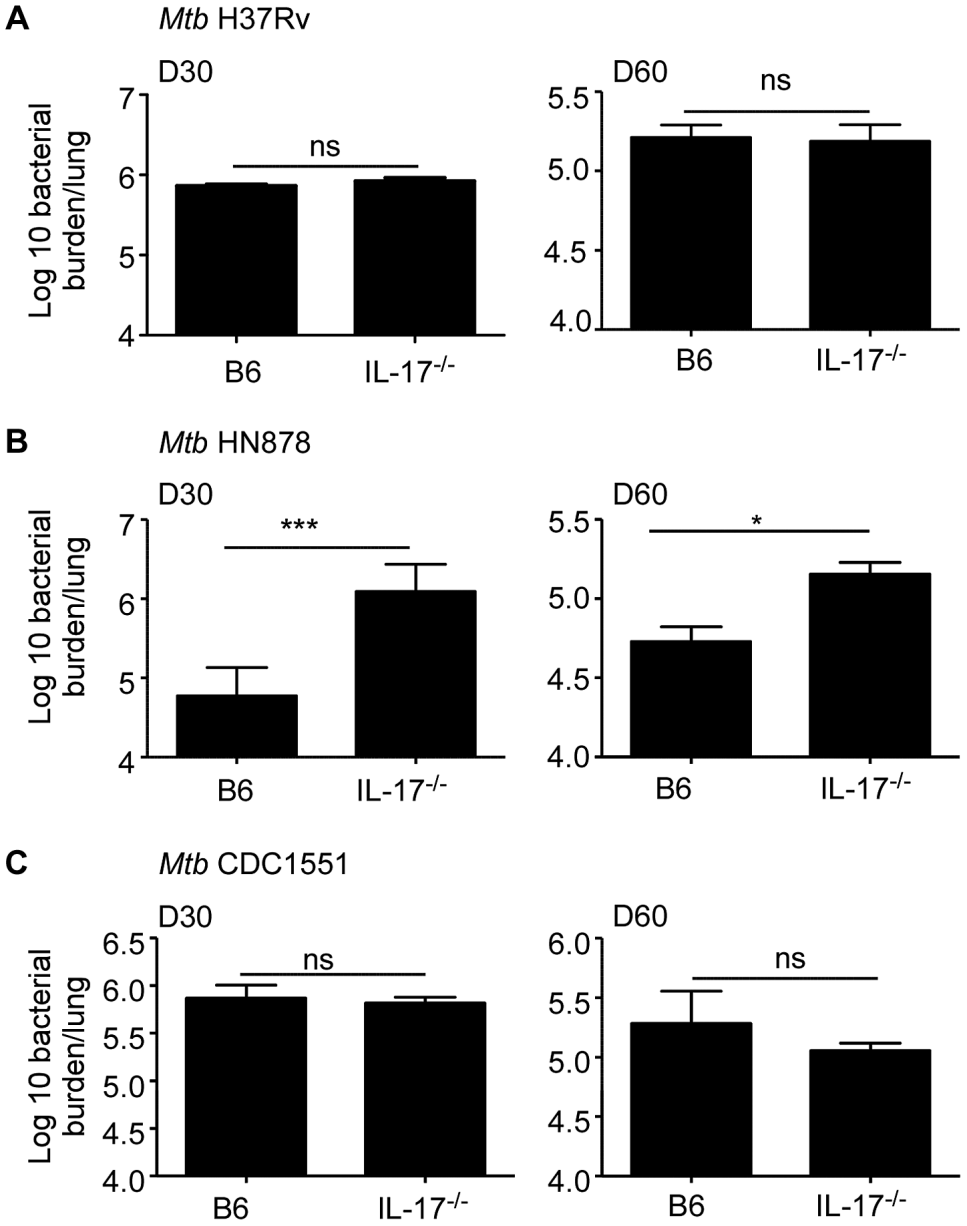
IL-17 is required for protective immunity against *Mtb* HN878 infection. B6 or IL-17^−/−^ mice were aerosol infected with ∼100 cfu *Mtb* H37Rv (a), *Mtb* HN878 (b) or *Mtb* CDC1551 (c). Lung bacterial burden was determined by plating on D30 or D60 post-infection. The data points represent the mean (±SD) of values from 3–5 samples. *p≤0.05, ***p≤0.0005, ns-not significant.

We had previously shown that IL-23 deficient mice (IL-23^−/−^) [13] and mice lacking IL-17 signaling (IL-17RA^−/−^) [14], [15] are not more susceptible than C57BL/6J mice (B6) mice, to low dose *Mtb* H37Rv aerosol infection. However, it is not known if the IL-17 pathway is required for protective immunity against infection with clinical isolates such as the hypervirulent *Mtb* HN878 strain. Thus, we aerosol infected B6 or mice lacking IL-17A (IL-17^−/−^), with low doses of either *Mtb* H37Rv or *Mtb* HN878 and determined the effect of IL-17 deficiency on bacterial burden in the lungs. As previously published with IL-17RA^−/−^ mice [14], [15], we found that the IL-17^−/−^ mice are not more susceptible than B6 mice to low dose aerosol *Mtb* H37Rv infection, at either early or later time points (Fig. 1A). Interestingly, when IL-17^−/−^ mice were infected with low doses of hypervirulent *Mtb* HN878 infection, we found that IL-17^−/−^ mice exhibited increased lung bacterial burden in the lungs at both early and later time points (Fig. 1B). To further test if this requirement for IL-17 in mediating protective immunity against clinical *Mtb* isolates was limited to *Mtb* HN878, or if IL-17 was required for protection against other clinical isolates, we infected B6 or IL-17^−/−^ mice with the less virulent clinical isolate *Mtb* CDC1551, and determined bacterial burden in the lungs. We found that similar to *Mtb* H37Rv infection, IL-17 was not required for protective immunity against *Mtb* CDC1551 infection, since IL-17^−/−^ mice exhibited comparable lung bacterial burden to B6 infected mice at both early and late time points (Fig. 1C). These data demonstrate for the first time that IL-17 is required for protective immunity against specific clinical *Mtb* isolates such as *Mtb* HN878, but not *Mtb* lab strains such as *Mtb* H37Rv, or less virulent clinical isolates such as *Mtb* CDC1551.

### *Mtb* HN878 infection induces potent IL-17 responses when compared to *Mtb* H37Rv infection

**Figure 2:**
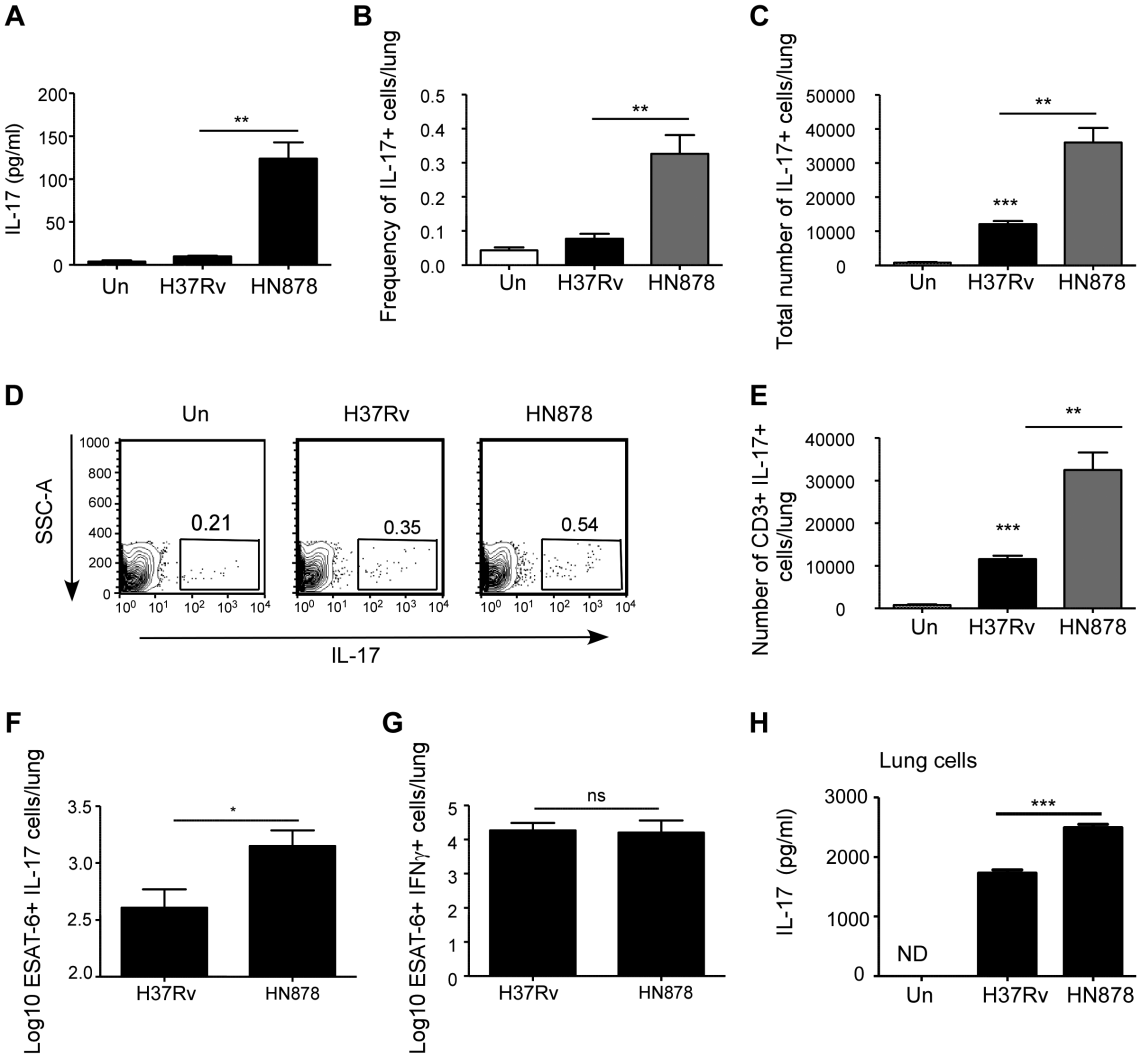
*Mtb* HN878 induces potent IL-17 responses in infected mice. B6 mice were either aerosol infected with ∼100 cfu *Mtb* H37Rv or *Mtb* HN878. Lungs were homogenized in saline on D30 post-infection and IL-17 protein levels were determined by Luminex (a). IL-17 GFP reporter mice were aerosol infected with ∼100 cfu *Mtb* H37Rv or *Mtb* HN878. The frequency (b) and absolute number (c) of lung IL-17-producing cells (GFP^+^) was determined by flow cytometry at D25 post-infection. Representative flow cytometry histograms of lung cell suspensions from uninfected (Un), *Mtb* H37Rv or *Mtb* HN878-infected IL-17 GFP reporter mice is shown (d). The number of CD3^+^IL-17^+^ lung cells was determined by flow cytometry (e). The percentage of ESAT-6_1–20_-specific, IL-17 (f) and IFN-γ (g) producing cells in the lungs of these mice was determined by ELISpot assay. 1×10^6^ lung CD11c^+^ cells from B6 mice and infected in vitro with *Mtb* H37Rv or *Mtb* HN878 (MOI 0.01). Subsequently, naïve B6 total lung cell suspensions were added to the culture, and 6 days later, IL-17 levels were determined in supernatants by ELISA (h). The data points represent the mean (±SD) of values from 3–5 samples. *p≤0.05, **p≤0.005, ***p≤0.0005. ns-not significant.

Our data suggest that IL-17 is required for early protective immunity following infection with *Mtb* HN878, but not *Mtb* H37Rv in mice. Thus, we next addressed if infection with *Mtb* H37Rv and HN878 induced different levels of IL-17 production in the lung. We found that lung IL-17 protein levels were significantly elevated in B6 mice infected with *Mtb* HN878, when compared to levels of IL-17 in lungs of B6 mice infected with *Mtb* H37Rv (Fig. 2A). In addition, when IL-17 reporter GFP mice were infected with similar low doses of either *Mtb* H37Rv or *Mtb* HN878, we found that *Mtb* HN878 infection induced a higher frequency and total number of IL-17-producing cells in the infected lungs (Fig. 2B–D). In addition, we found that the majority of the lung IL-17^+^ cells were CD3^+^ T cells (Fig. 2E). Furthermore, we found that increased numbers of *Mtb* antigen-specific ESAT6_1–20_ IL-17-producing cells were detected in *Mtb* HN878-infected lungs, when compared to *Mtb* H37Rv-infected lungs (Fig. 2F). In contrast, no significant differences were observed in the number of ESAT-6–specific IFN-γ-producing cells in the lungs of B6 mice infected with either *Mtb* HN878 or *Mtb* H37Rv (Fig. 2G). Differentiation of Th17 cells is dependent on instructive signals provided by APCs, thus we infected lung CD11c^+^ cells in vitro with the two strains of *Mtb*, and subsequently co-cultured them with naïve lung cells for 6 days. Increased levels of IL-17 were detected in *Mtb* HN878-infected co-culture supernatants, when compared to levels detected in supernatants from *Mtb* H37Rv-infected co-cultures (Fig. 2H). These data together suggest that *Mtb* HN878 strain induces higher levels of IL-17 production in lung cells, when compared to *Mtb* H37Rv infection.

### Enhanced IL-17 production following *Mtb* HN878 infection is TLR-2 and IL-1β dependent

**Figure 3:**
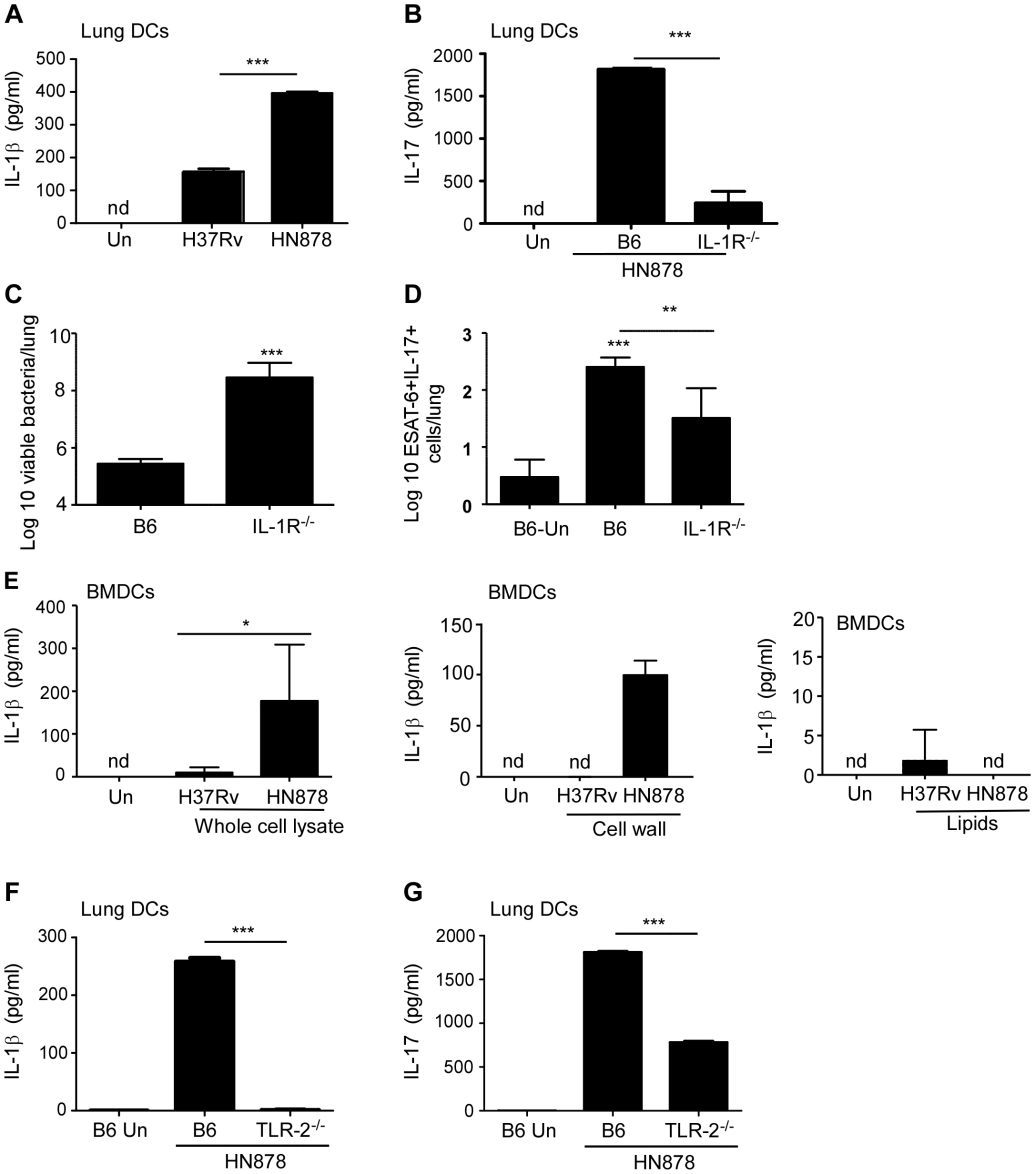
*Mtb* HN878-driven IL-17 production is dependent on TLR-2 and IL-1β. 1×10^6^ lung DCs from B6 mice were infected in vitro with *Mtb* H37Rv or *Mtb* HN878 (MOI 0.01) for 48 h and IL-1β production was determined in the supernatants (a). 1×10^6^ B6 DCs were infected with *Mtb* H37Rv or *Mtb* HN878 (MOI 0.01), following which naïve B6 or IL-1R−/− total lung cell suspensions were added to the culture, and IL-17 levels were determined by ELISA on day 6 of the co-culture (b). B6 or IL-1R^−/−^ mice were aerosol infected with ∼100 cfu *Mtb* HN878 and lung bacterial burden was determined on D30 post-infection (c). The number of ESAT-6_1–20_-specific, IL-17-producing cells in the lungs of uninfected (Un), *Mtb* HN878-infected B6 and IL-1R^−/−^ mice was determined by ELISpot assay (d). 1×10^6^ BMDCs were treated in vitro with whole cell lysate, cell wall extract or a lipid extract (20 µg/ml) of *Mtb* H37Rv or *Mtb* HN878, and IL-1β production in cell culture supernatants was determined by ELISA (e). 1×10^6^ lung CD11c^+^ cells from B6 or TLR-2^−/−^ mice and infected in vitro with *Mtb* H37Rv or *Mtb* HN878. Subsequently, naïve B6 or TLR-2^−/−^ total lung cell suspensions were added to the culture, and IL-1β (f) and IL-17 (g) levels were determined by ELISA. The data points represent the mean (±SD) of values from 3–5 samples. *p≤0.05, **p≤0.005, ***p≤0.0005. nd-not detected.

In order to define the mechanism driving enhanced IL-17 production during *Mtb* HN878 infection, we analyzed induction of polarizing cytokines by lung DCs following infection with *Mtb* H37Rv and *Mtb* HN878. We found increased IL-1β levels in supernatants of lung DCs infected with *Mtb* HN878 strain, but not *Mtb* H37Rv (Fig. 3A). IL-1β has a critical role in IL-17 production [25]. Importantly, when lung DCs were infected with *Mtb* HN878, and IL-1R^−/−^ lung cells were co-cultured with infected DCs, IL-17 production was significantly impaired in culture supernatants (Fig. 3B), demonstrating that IL-17 production is IL-1β pathway dependent. To confirm our in vitro findings in vivo during *Mtb* infection, we infected B6 or IL-1R^−/−^ mice with low doses of aerosolized *Mtb* HN878 and found that IL-1R^−/−^ mice had significantly higher bacterial burden (Fig. 3C), and this coincided with significantly reduced induction of *Mtb*-specific IL-17-producing T cells (Fig. 3D). Together, these data demonstrate that *Mtb* HN878-driven IL-17 production is IL-1β dependent, both in vitro and in vivo.

To further define the mechanism underlying IL-1β induction by *Mtb* HN878, we incubated bone marrow derived DCs (BMDCs) with different subcellular preparations from *Mtb* and found that treatment with *Mtb* HN878 whole cell lysates and cell wall extracts, but not lipids from *Mtb* HN878, induced IL-1β in BMDCs (Fig. 3E). Given that TLR-2 recognizes cell wall components from *Mtb* [26], and that its activation has been linked to IL-1β production [27], we next determined whether IL-1β and IL-17 production were dependent on activation through TLR-2. We found that *Mtb* HN878-infection of TLR-2^−/−^ lung DCs, had a dramatic impairment in IL-1β production when compared to infection of B6 DCs (Fig. 3F), and coincided with low IL-17 production in DC: lung cell co-cultures (Fig. 3G). These data together suggest that when compared to *Mtb* H37Rv infection, *Mtb* HN878 infection induces higher levels of IL-1β production in APCs through a TLR-2-dependent mechanism, and this drives increased IL-17 production.

### IL-17 mediates protection against *Mtb* HN878 infection by driving CXCL-13 induction, lung lymphoid follicle formation and macrophage activation

IL-17 is well documented to drive the production of molecules such as Keratinocyte chemoattractant (KC), and Granulocyte Colony Stimulating Factor (G-CSF) to mediate neutrophil recruitment and accumulation [12]. However, we did not find any defects in total lung neutrophil numbers and lung neutrophil accumulation within granulomas of B6 or IL-17^−/−^ *Mtb* HN878 infected mice (data not shown). In addition, we did not find any differences in pulmonary levels of KC and G-CSF in B6 and IL-17^−/−^ *Mtb* HN878 infected mice (data not shown). These data together suggest the role for IL-17 in protective immunity against *Mtb* HN878 infection is not mediated through its well documented role in neutrophil recruitment. Since IL-17 can drive the induction of Th1 responses in some intracellular pulmonary bacterial infection models [28], [29], we next determined if there were any defects in generation of Th1 immune responses. Interestingly, we did not find any differences in the accumulation of IFN-γ (Fig. S1A) or TNF-α-producing (Fig. S1B) *Mtb*-specific T cells in the lungs of B6 and IL-17R^−/−^ *Mtb* HN878-infected mice. In addition, we also found comparable numbers of activated CD4^+^ T cells accumulating in the *Mtb* HN878-infected B6 and IL-17R^−/−^ lungs (Fig. S1C), including total IFN-γ (Fig. S1D) and IL-2-producing T cells (Fig. S1E). These data suggest that the generation and accumulation of activated Th1 responses are not defective in mice deficient in the IL-17 pathway.

**Figure 4:**
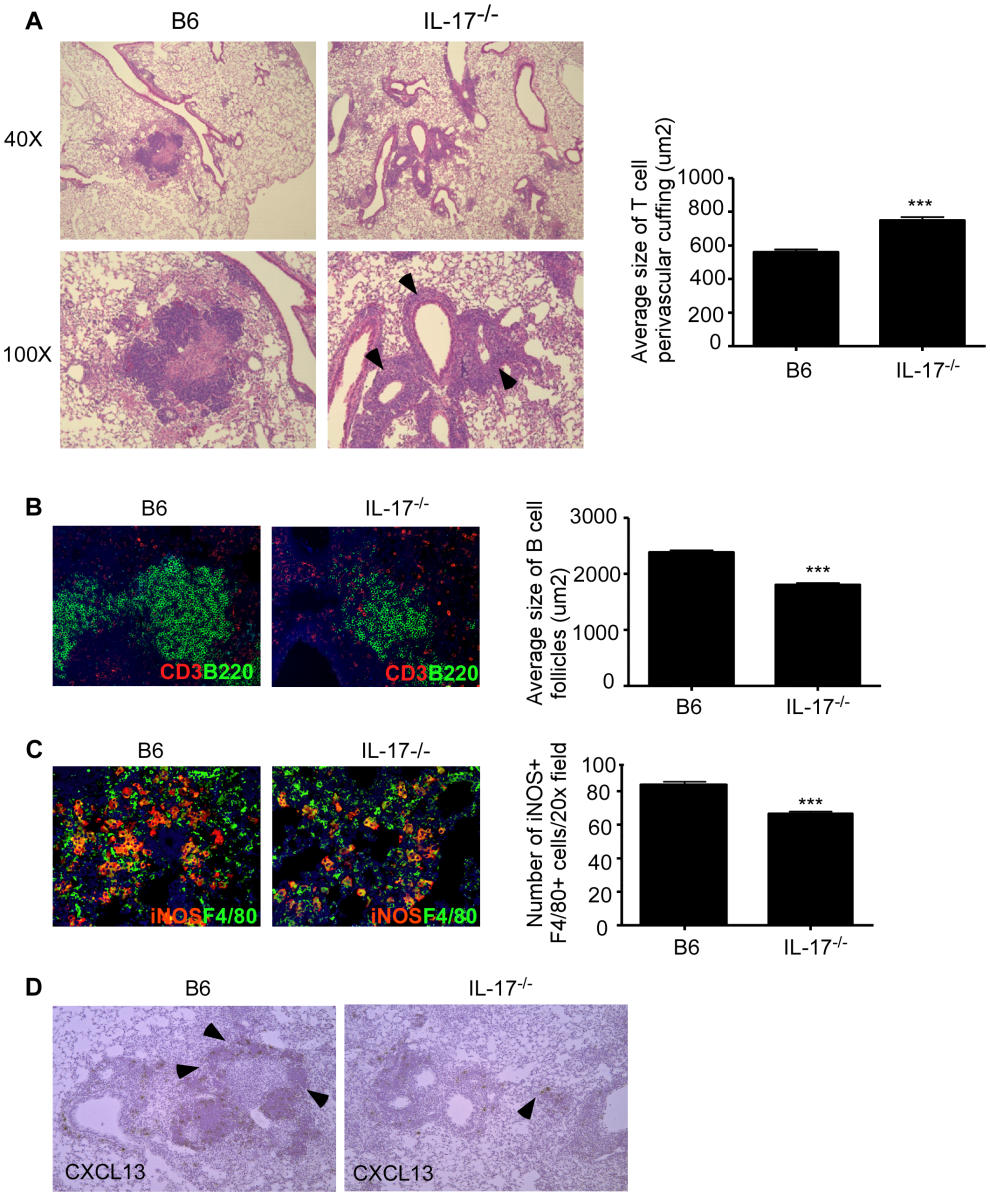
IL-17 mediates control of *Mtb* HN878 through CXCL13 induction, lymphoid follicle formation and macrophage activation. B6 and IL-17^−/−^ mice were aerosol infected with ∼100 cfu *Mtb* HN878 and lungs were harvested on D60 post-infection. Pulmonary histology was assessed on formalin-fixed, paraffin embedded lung sections that were stained with H&E (a). Arrows depict T cell perivascular cuffing. Serial sections from infected lungs were also processed for immunofluorescence using antibodies specific for CD3 and B220 (b) or iNOS and F4/80 (c), and stained for CXCL13 mRNA expression by in situ hybridization (d). 400× magnification for immunofluorescence images. Arrows within (d) indicate CXCL-13 mRNA expression. The average size of T cell perivascular cuffing (a) and B cell lymphoid follicles (b) was calculated using the morphometric tool of the Zeiss Axioplan microscope. The number of iNOS-expressing cells per field (c) was counted and shown. n = 5–9 mice per group. ***p≤0.005.

We have recently described a role for early vaccine-induced IL-17 in mediating CXCL-13 expression which resulted in localization of CXCR5^+^ cytokine producing T cells near *Mtb*-infected macrophages, an event crucial for optimal *Mtb* control [30]. The correct localization of T cells expressing CXCR5 within the lung parenchyma, results in formation of ectopic lymphoid structures, which is required for activation of infected macrophages for control of *Mtb* [30]. Thus, we next addressed whether the increased susceptibility in IL-17^−/−^ mice infected with *Mtb* HN878 was due to defects in T cell localization near *Mtb*-infected macrophages within the lung. We found that IL-17^−/−^ mice infected with *Mtb* HN878 exhibited increased lung perivascular T cuffing (Fig. 4A), and this coincided with poorly formed lymphoid follicles within the lungs of IL-17^−/−^ *Mtb* HN878-infected mice, when compared to B6 *Mtb* HN878-infected mice (Fig. 4B). Importantly, this coincided with decreased accumulation of activated iNOS-producing macrophages within the IL-17^−/−^ *Mtb* HN878-infected lung inflammatory lesions, when compared to the B6 *Mtb* HN878-infected lesions (Fig. 4C). To exclude a direct effect of IL-17 on macrophage activation and *Mtb* control, bone marrow macrophages (BMDMs) from B6 mice were infected with *Mtb* HN878 and treated with either IFN-γ or IL-17, or both cytokines. Although we found that IL-17 treatment induced some iNOS production in uninfected macrophages, it did not enhance iNOS production in *Mtb* HN878-infected macrophages or enhance control of *Mtb* in infected macrophages (Fig. S2A–B). As expected, IFN-γ treatment resulted in increased iNOS production by macrophages and improved bacterial control, when compared to untreated macrophages or IL-17 treated *Mtb*-infected macrophages (Fig. S2A–B). Instead, we found that coincident with reduced lymphoid follicle formation in IL-17^−/−^ *Mtb* HN878-infected lungs, expression of CXCL-13 mRNA expression within lung lymphoid follicles was also reduced (Fig. 4D). These data together suggest that following *Mtb* HN878 infection, enhanced IL-17 production driven by the infection, plays a role in induction of CXCL-13, localization of cytokine producing T cells near *Mtb*-infected macrophages, and *Mtb* control.

### Overexpression of IL-17 restores protective immunity in IL-17^−/−^ mice challenged with *Mtb* HN878

**Figure 5:**
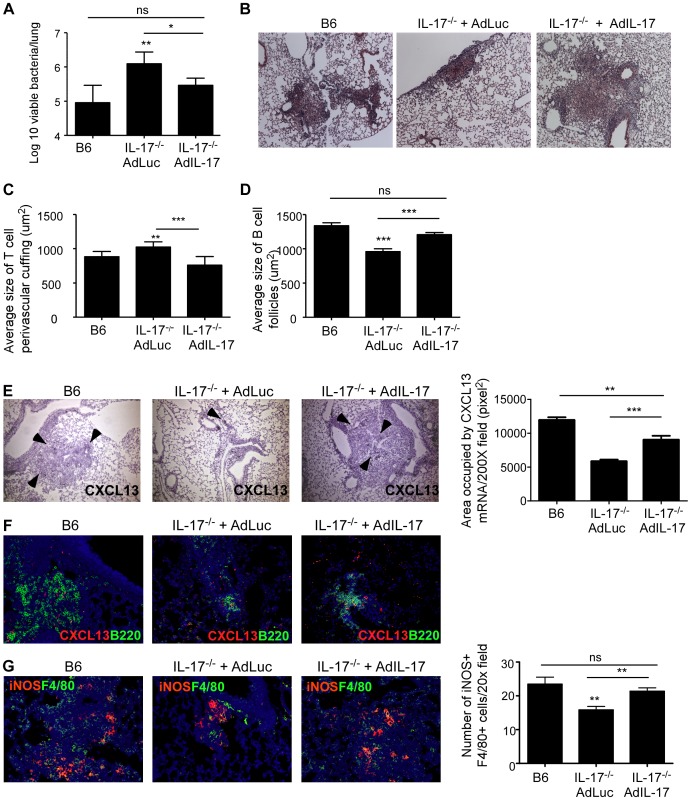
Adenoviral IL-17 overexpression reverses the increased susceptibility to *Mtb* HN878 infection in IL-17^−/−^ mice. B6 or IL-17^−/−^ mice were aerosol infected with ∼100 cfu *Mtb* HN878 and were either infected with a vector control adenovirus expressing luciferase (Adluc) or with an adenoviral vector overexpressing IL-17 (AdIL-17) on day 9. Lung bacterial burden was determined on D30 post-*Mtb* infection (a). Pulmonary histology was assessed on formalin-fixed, paraffin embedded lung sections that were stained with H&E (b). 100× magnification for H&E sections. The average size of T cell perivascular cuffing (c) and B cell lymphoid follicles (d) was calculated using the morphometric tool of the Zeiss Axioplan microscope. CXCL13 mRNA expression in formalin fixed, paraffin embedded lung sections was studied by in situ hybridization (e). 100× magnification, arrows indicate areas of CXCL-13 mRNA expression. The area occupied by CXCL13 signal by in situ hybridization per 200× field was calculated using the morphometric tool of the Zeiss Axioplan microscope. Serial sections from infected lungs were also processed for immunofluorescence using antibodies specific for CXCL13 and B220 (f). 400× magnification. Immunofluorescence staining was also performed using antibodies specific for or iNOS and F4/80 and the numbers of iNOS^+^ cells were counted (g). 400× magnification. The data points represent values from n = 5 mice per group. *p≤0.05, **p≤0.005, ***p≤0.0005, ns-not significant.

**Figure 6:**
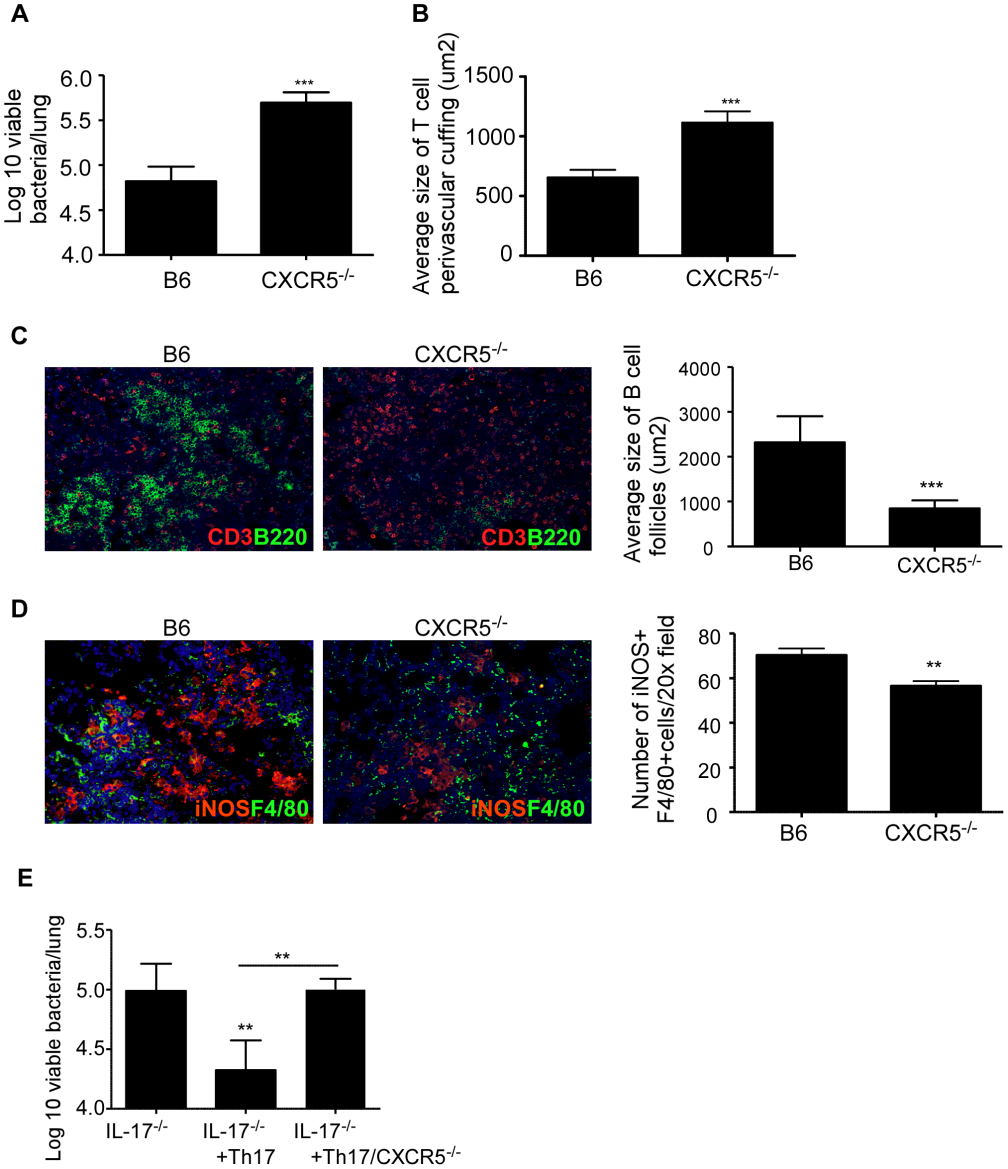
CXCR5 deficiency increases susceptibility to *Mtb* HN878 infection. B6 and CXCR5^−/−^ mice were aerosol infected with ∼100 cfu *Mtb* HN878 and lung bacterial burden was determined on D30 post-infection (a). Pulmonary histology was assessed on formalin-fixed, paraffin embedded lung sections. The average size of T cell perivascular cuffing (b) and B cell lymphoid follicles (c) was calculated using the morphometric tool of the Zeiss Axioplan microscope. Serial sections from infected lungs were also processed for immunofluorescence using antibodies specific for CD3 and B220 (c) or iNOS and F4/80 (d). The number of iNOS-expressing cells per field (d) was counted and shown. 400× magnification. Wild type or CXCR5^−/−^ ESAT-6 TCR Tg CD4^+^ T cells were in vitro differentiated to the Th17 subset and 2×10^6^ Th17 cells were adoptively transferred into IL-17^−/−^ mice and then infected with low doses of *Mtb* HN878. Lung bacterial burden was determined on D30 post-infection (e). The data points represent values from n = 5 mice per group. **p≤0.005, ***p≤0.0005.

Our data show that IL-17 is required for protective immunity against *Mtb* HN878 infection by mediating correct T cell localization near *Mtb*-infected macrophages for optimal macrophage activation. We therefore tested if over expression of IL-17 by adenoviral vectors would rescue the increased susceptibility observed in IL-17^−/−^ *Mtb* HN878 infected mice. Thus, IL-17^−/−^ *Mtb* HN878 infected mice received a single intratracheal delivery of either adenovirus expressing a luciferase control vector or adenovirus expressing IL17. We found that IL-17 overexpression reversed the increased bacterial burden seen in IL-17^−/−^ *Mtb* HN878 infected mice (Fig. 5A), resulted in decreased T cell perivascular cuffing (Fig. 5B,C), improved formation of lung lymphoid follicles (Fig. 5D), and coincident increase in CXCL-13 mRNA expression (Fig. 5e) and CXCL-13 protein (Fig. 5F), within B cell lymphoid follicles in the lung. As a result, IL-17 overexpression resulted in increased accumulation of iNOS-expressing macrophages within the inflammatory lesions (Fig. 5G). To further confirm that CXCL-13 expression is critical for localization of CXCR5-expressing Th1 cells within the lymphoid follicles and *Mtb* control, we infected CXCR5^−/−^ mice with *Mtb* HN878. We found that CXCR5^−/−^ mice exhibited increased bacterial burden when compared to B6 *Mtb* HN878 infected mice (Fig. 6A). Importantly, we found that CXCR5^−/−^ *Mtb* infected mice demonstrated enhanced T cell perivascular cuffing (Fig. 6B) and this coincided with reduced formation of lymphoid follicles (Fig. 6C), measured by determining the average area of B cell follicle within the lungs of B6 and CXCR5^−/−^ mice. In addition, we found that the impaired localization of T cells within the lung parenchyma resulted in decreased accumulation of iNOS-producing macrophages (Fig. 6D), within the lung inflammatory lesions, suggesting sub-optimal activation of macrophages for *Mtb* control. In order to mechanistically address if presence of Th17 cells could rescue the increased susceptibility seen in IL-17^−/−^ mice, we adoptively transferred *Mtb*-specific Th17 cells generated from wild type ESAT-6 TCR Tg mice or from CXCR5^−/−^ ESAT-6 TCR Tg mice, into IL-17^−/−^ mice which were then infected with aerosolized *Mtb* HN878. Consistent with a role for IL-17 in mediating protection against *Mtb* HN878 control, adoptive transfer of wild type Th17 cells significantly reduced the bacterial burden (Fig. 6E). In contrast, adoptive transfer of Th17 cells derived from CXCR5^−/−^ mice did not decrease lung bacterial burden (Fig. 6E). These data together conclusively provide evidence that IL-17 expression during *Mtb* HN878 infection is required for effective induction of CXCL-13, a chemokine that is key to facilitating productive interactions between cytokine producing CXCR5-expressing T cells and *Mtb*-infected macrophages for optimal *Mtb* HN878 control in the lung.

### IL-17R expression on non-hematopoietic cells is crucial for mediating protective immunity against *Mtb* HN878 infection

**Figure 7:**
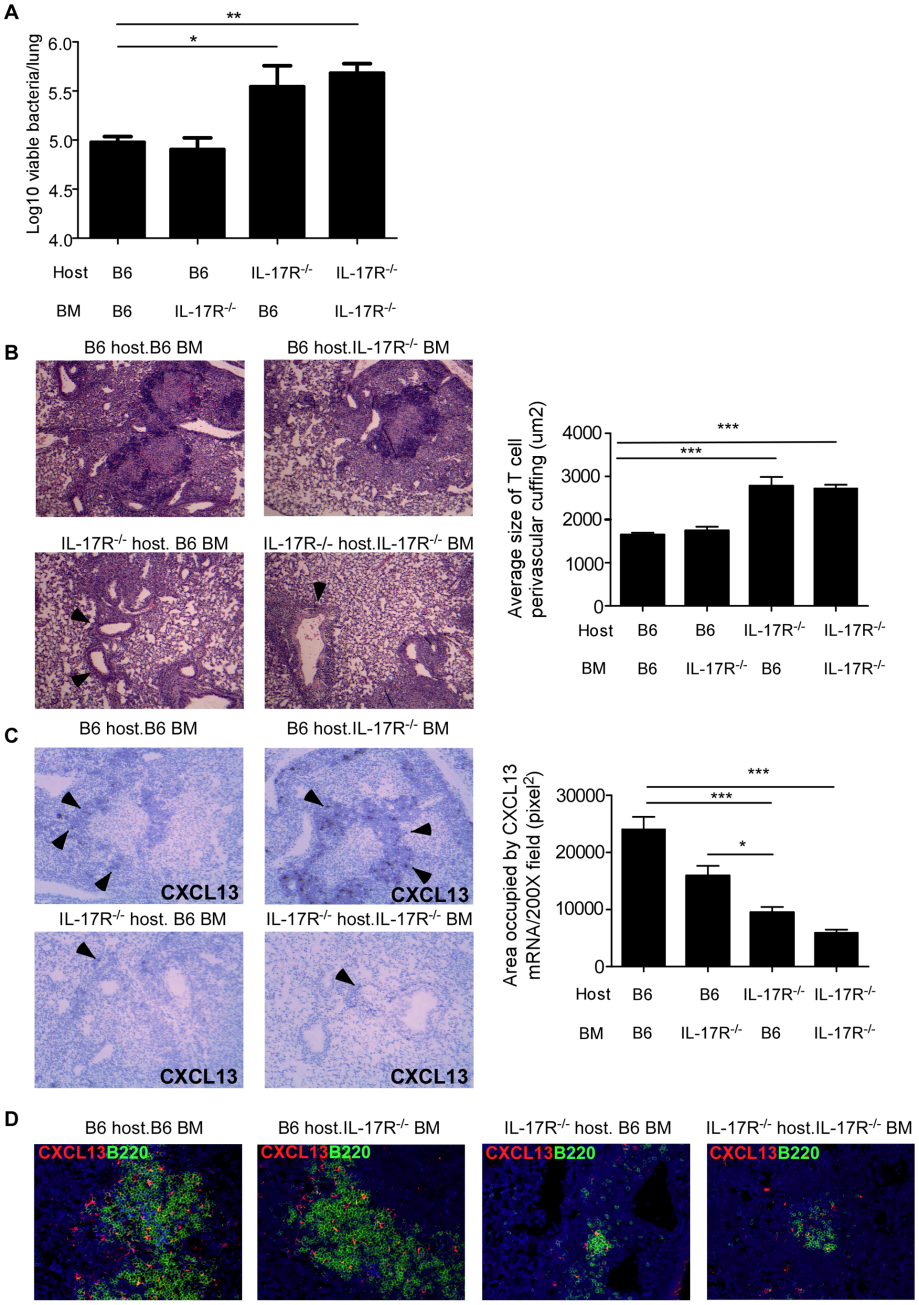
IL-17R expression on non-hematopoietic cells is required for protective immunity against *Mtb* HN878. Hematopoietic IL-17R^−/−^ BMC mice (B6 host/−/− BM), non-hematopoietic IL-17R^−/−^ BMC mice (−/− host/B6 BM), complete IL-17R^−/−^ BMC mice (−/− host/−/−BM) and complete B6 BMC mice (B6 host/B6 BM) were generated as described under methods. BMC mice were aerosol infected with ∼100 cfu *Mtb* HN878 and lung bacterial burden was determined on D30 post-infection (a). Pulmonary histology was assessed on formalin-fixed, paraffin embedded lung sections that were stained with H&E and the average size of T cell perivascular cuffing was calculated using the morphometric tool of the Zeiss Axioplan microscope (b). T cell perivascular cuffing is indicated by the arrows. CXCL13 mRNA expression in formalin fixed, paraffin embedded lung sections was studied by in situ hybridization (c). 100× magnification, arrows point to typical areas of CXCL13 mRNA expression. The area occupied by CXCL13 signal by in situ hybridization per 200× field was calculated using the morphometric tool of the Zeiss Axioplan microscope. Serial sections from infected lungs were also processed for immunofluorescence using antibodies specific for CXCL13 and B220 (d). 400× magnification. The data points represent values from n = 3–5 mice per group. *p≤0.05, **p≤0.005.

IL-17R is primarily expressed on non-hematopoietic cells, but IL-17R can also be expressed on hematopoietic cells such as macrophages and dendritic cells [12]. Thus, we next addressed if IL-17R signaling on hematopoietic or non-hematopoietic cells was essential for inducing CXCL-13 expression and mediating protection against *Mtb* HN878 infection. Thus, we generated hematopoietic IL-17R^−/−^ bone marrow chimeric (BMC) mice (B6 host/−/− BM) or non-hematopoietic IL-17R^−/−^ BMC mice (−/− host/B6 BM) and infected with *Mtb* HN878. As expected based on our data, complete IL-17R^−/−^ BMC mice (−/−host/−/− BM) were more susceptible to *Mtb* HN878 infection than complete B6 BMC mice (B6 host/B6 BM) (Fig. 7A), and demonstrated defects in T cell localization (Fig. 7B), formation of lymphoid follicles and CXCL-13 mRNA expression (Fig. 7C), and CXCL-13 protein expression within B cell lymphoid follicles (Fig. 7D). Interestingly, non-hematopoietic, but not hematopoietic IL-17R^−/−^ BMC mice had increased lung bacterial burden (Fig. 7A), and this coincided with increased perivascular T cell cuffing (Fig. 7B) and reduced CXCL-13 mRNA and protein expression (Fig. 7C,D). These data clearly demonstrate that IL-17 signaling in non-hematopoietic cells is required for CXCL-13 induction, to mediate correct T cell localization near *Mtb* infected macrophages to confer protective immunity against *Mtb* HN878 infection.

## Discussion

The *Mtb* isolate HN878, belongs to the W-Beijing family of isolates, which have been associated with outbreaks throughout the world, and with clusters of drug-resistant disease in the United States [31]. W-Beijing strains have significant clinical relevance because they are over-represented among drug resistant *Mtb* isolates, and are associated with HIV infection in humans [7]. In animal models, infection is considered hypervirulent due to increased immunopathology and mortality [8]. In the current paper, we show that IL-17 is required for early protective immunity against *Mtb* HN878 infection, but not lab adapted *Mtb* isolates such as H37Rv, or less virulent *Mtb* clinical isolates such as CDC1551. Our data also suggest that the dependence on IL-17 to drive early protective immunity against *Mtb* HN878 is due to the differential ability of *Mtb* HN878 to induce high levels of IL-17 production, through an IL-1β-TLR-2 dependent pathway. Thus, defining the differential immune requirements that mediate protective immunity against clinical isolates such as the widely spreading W-Beijing isolate, is critical for successful design of vaccines against emerging strains of *Mtb*. Thus, our novel results demonstrating a role for IL-17 in primary immunity to specific *Mtb* strains, have far reaching implications for the future design of vaccines and therapies to prevent and treat TB worldwide, especially emergent strains of clinical relevance for public health.

Early studies using animal models showed that infection with *Mtb* HN878 induced Type I interferons, and this coincided with decreased induction of proinflammatory cytokines such as TNF-α, IFN-γ and IL-2, reduced T cell activation and increased susceptibility to infection [8], [10], [11]. The hypervirulent phenotype of *Mtb* HN878 was initially linked to production of phenolic glycolipids (PGL), one of the major lipid components of the mycobacterial cell wall, that could be mediating the inhibition of protective Th1 responses [8], [10], [11]. Interestingly, when PGL was expressed in *Mtb* H37Rv, a strain normally devoid of PGL synthesis, it did not lead to increased virulence in infected mice and rabbits, suggesting that PGLs in concert with other bacterial factors likely mediates the hypervirulence of W-Beijing *Mtb* strains [32]. Furthermore, it has been recently shown that PGL enhances infectivity through CCR2-mediated TLR-independent recruitment of permissive macrophages at the earliest stages of infection, suggesting the presence of PGL in *Mtb* HN878 as a likely factor contributing to the increased transmission of *Mtb* HN878 [33]. In contrast, in the current study, we show that DCs infected with *Mtb* HN878 are stimulated likely by a cell wall component that binds TLR2, and triggers the secretion of IL-1β. In addition, the increased induction of IL-1β in infected DCs mediates the induction of IL-17 production, primarily in CD3^+^ T cells. Accordingly, our studies show that in the presence of low IL-17 induction following *Mtb* H37Rv infection, absence of IL-17 as shown here, or absence of IL-17R as published before [14], [15], does not impact protective immunity. In contrast, consistent with the increased induction of IL-17 seen in *Mtb* HN878 infected lungs, IL-17 is required for early protective immunity against infection with *Mtb* HN878. Our data also show that a protective role for IL-17 is not observed for all clinical *Mtb* isolates, as IL-17^−/−^ mice infected with the less virulent clinical isolate *Mtb* CDC1551, does not show early increased susceptibility to infection. Our data described here has tested the early protective role for IL-17 in *Mtb* infection, and future studies determining whether IL-17 is required for maintenance of *Mtb* control during chronic stages of *Mtb* infection will be important. Without doubt, future studies focused on defining whether specific lineages of *Mtb* require IL-17 for protective immunity in acute and chronic *Mtb* infection will be critical for successful vaccine design for global TB control, by tailoring more specific strategies for prevalent emerging strains of *Mtb* in certain geographical areas.

Our recent work has put forth the new “working hypothesis” that a protective TB granuloma in the lung is predominantly composed of lymphoid follicles, where T cells and B cells are strategically positioned near infected macrophages to form lymphoid follicles or optimal activation to control *Mtb* infection [17], [20]. IL-17 induces expression of CXCL-13 in the lung and mediates generation of lymphoid follicles following inflammation [17], [34]. Consistent with this newly described role for IL-17 in driving formation of lymphoid follicles in the lung, our data show that IL-17^−/−^ mice which were more susceptible to *Mtb* HN878 infection have significant defects in T cell localization and formation of lung lymphoid follicles within TB granulomas. In addition, we found that the poorly formed lymphoid follicles with the lungs of IL-17^−/−^ mice exhibited reduced induction of CXCL13 mRNA and protein. Our data show that IL-17^−/−^ *Mtb* HN878-infected lungs accumulate similar numbers of proinflammatory T cells producing IFN-γ, IL-2 and TNF-α, suggest that the increased susceptibility to infection is not due to defects in generation, or accumulation of proinflammatory T cells in the lung, but due to defects in localization of cytokine-producing T cells within lymphoid follicles to mediate macrophage activation. The fact that overexpression of IL-17 using adenoviral vectors can reverse the increased susceptibility, CXCL-13 expression, T cell localization and lymphoid follicle formation within the IL-17^−/−^ *Mtb* HN878-infected lung, further supports and validates this hypothesis. However, it is also possible that absence of IL-17 impacts the generation and accumulation of B cells or other T cells such as CD8^+^ T cells to mediate formation of B cell lymphoid follicles, and should be addressed in future studies. IL-17R^−/−^ mice infected with *Mtb* H37Rv exhibit a transient early defect in lymphoid follicle formation, coinciding with absence of a role for IL-17 pathway in mediating protection [15]. These data together suggest that following infection with *Mtb* HN878, IL-17 has a non-redundant and prominent role to play in induction of CXCL-13 expression, likely through its effects on non-hematopoietic cells such as epithelial cells and fibroblasts. This is consistent with the ability of IL-17 to drive CXCL-13 expression in fibroblasts in vitro [15]. Interestingly, both CXCL-13 and CXCR5^−/−^ mice are equally susceptible to infection with either *Mtb* H37Rv [20], [23] or *Mtb* HN878 described here, implicating IL-17-independent pathways in CXCL-13 induction during *Mtb* H37Rv infection. Further studies delineating and characterizing common versus differential protective correlates across different *Mtb* lineages will be necessary in designing vaccines, specific for clinical strains that are endemic in different geographical locations of the world.

Our recent studies using CXCR5^−/−^ mice in *Mtb* H37Rv infection model, have demonstrated that expression of CXCR5 is required for T cell localization within the lung and macrophage activation for *Mtb* control [20]. Importantly, we showed that adoptive transfer of CD4^+^ T cells expressing CXCR5 into *Mtb* H37Rv-infected CXCR5^−/−^ mice, was sufficient to allow T cells to localize within the lung, reverse generation of lymphoid tissues and improve disease outcome [20]. Consistent with these recent findings, we report here that CXCR5^−/−^ mice when infected with *Mtb* HN878 are also susceptible to *Mtb* HN878 infection, demonstrate defects in localization of T cells, formation of lymphoid follicles and activation of macrophages within TB granulomas. Importantly, IL-17^−/−^ mice that receive *Mtb*-specific Th17 cells expressing CXCR5, could reverse the increased susceptibility and associated disease phenotype, but adoptive transfer of *Mtb*-specific CXCR5^−/−^ Th17 cells could not rescue the disease phenotype in IL-17^−/−^ *Mtb* HN878-infected mice. These data together mechanistically provide evidence that following *Mtb* HN878 infection, cytokine producing CD4^+^ T cells express CXCR5 and respond to signals from IL-17-dependent CXCL-13 to localize within the lung parenchyma to form lymphoid follicles, and activate macrophages for *Mtb* control.

IL-17 is generally thought to be required for protective immunity against extracellular pathogens, by inducing chemokines that drive neutrophil recruitment for pathogen control [12]. Thus, it was not surprising that early studies demonstrated that IL-17 was not required for protective immunity against intracellular pathogen such as *Mycobacteria* [13], [14], [35], *Listeria* [14] and *Salmonella* infections [36]. However, more recent work by us and others has demonstrated that in some models of intracellular infections, IL-17 is required for protective immunity to drive the induction of IL-12 and generate Th1 responses [28], [29]. In contrast, the more widely appreciated role for IL-17 in TB, is its role in mediating vaccine-induced protection against *Mtb* challenge [16], [17]. Accordingly, targeting IL-17 to improve vaccine design for TB is an active avenue of research [37]. Thus, our new data demonstrating that the IL-17 pathway also plays a role in primary immunity following infection with *Mtb* HN878, significantly changes the existing paradigm that IL-17 is not required for primary immunity against TB. Interestingly, some human studies report increased IL-17 in active TB patients [38], [39], while other studies report increased IL-17 in latent TB patients [40], [41] and healthy controls [41], [42], suggesting IL-17 may be associated with either inflammation or protection, respectively. Thus, differential IL-17 expression by some emerging *Mtb* strains may define the final role for IL-17 in protection or pathology during TB, and needs to be carefully studied.

In summary, data presented here demonstrates that IL-17 has an early protective role to play in immunity against *Mtb* HN878 infection, by mediating induction of chemokines, T cell localization within lymphoid follicle for macrophage activation and *Mtb* control. These results demonstrate a novel and previously undescribed role for IL-17 in primary immunity to TB, especially considering that W-Beijing strains such as *Mtb* HN878 are emerging as major drug resistant *Mtb* strains that are not protected by prior BCG vaccination. Thus, targeting IL-17 to improve vaccine stategies for protection against emerging *Mtb* strains such as W-Beijing *Mtb* strains, may prove critical in controlling global TB burdens.

## Supporting Information

Figure S1 **IL-17R^−/−^ mice have unaltered Th1 responses.** B6 or IL-17R^−/−^ mice were aerosol infected with ∼100 cfu *Mtb* HN878 and lungs were collected on D30 post-infection. The percentage of ESAT-6_1–20_-specific, IFN-γ (a) and TNF-α (b)-producing cells in the lungs of *Mtb*-infected mice and uninfected mice (Un) was determined by *Mtb*-specific ELISpot assay. The total number of activated lung CD4^+^ T cells (CD3^+^CD4^+^CD44^+^) (c) that produced IFN-γ (d) or IL-2 (e) was determined by flow cytometry. The data points represent values from n = 3–5 mice per group *p≤0.05, **p≤0.005, ***p≤0.0005. ns-not significant.
(TIF)

Figure S2 **IL-17 does not directly mediate macrophage activation and** ***Mtb*** **control.** BMDMs were in vitro infected with *Mtb* HN878 (MOI 5) and treated in the presence of IL-17, IFN-γ or both cytokines, and macrophage-mediated killing was determined by bacterial plating of macrophage lysates (a). Nitrite production by these macrophages in culture supernatants was determined by using Griess reaction (b). The data points represent the mean (±SD) of values from 3–5 samples. ***p≤0.0005. ns-not significant.
(TIF)
