# Supplementary figures and images for: Unexpected Role for IL-17 in Protective Immunity against Hypervirulent Mycobacterium tuberculosis HN878 Infection
Source: PLoS Pathog. 2014 May 15;10(5):e1004099. doi: 10.1371/journal.ppat.1004099 (PMC4022785; doi:10.1371/journal.ppat.1004099)

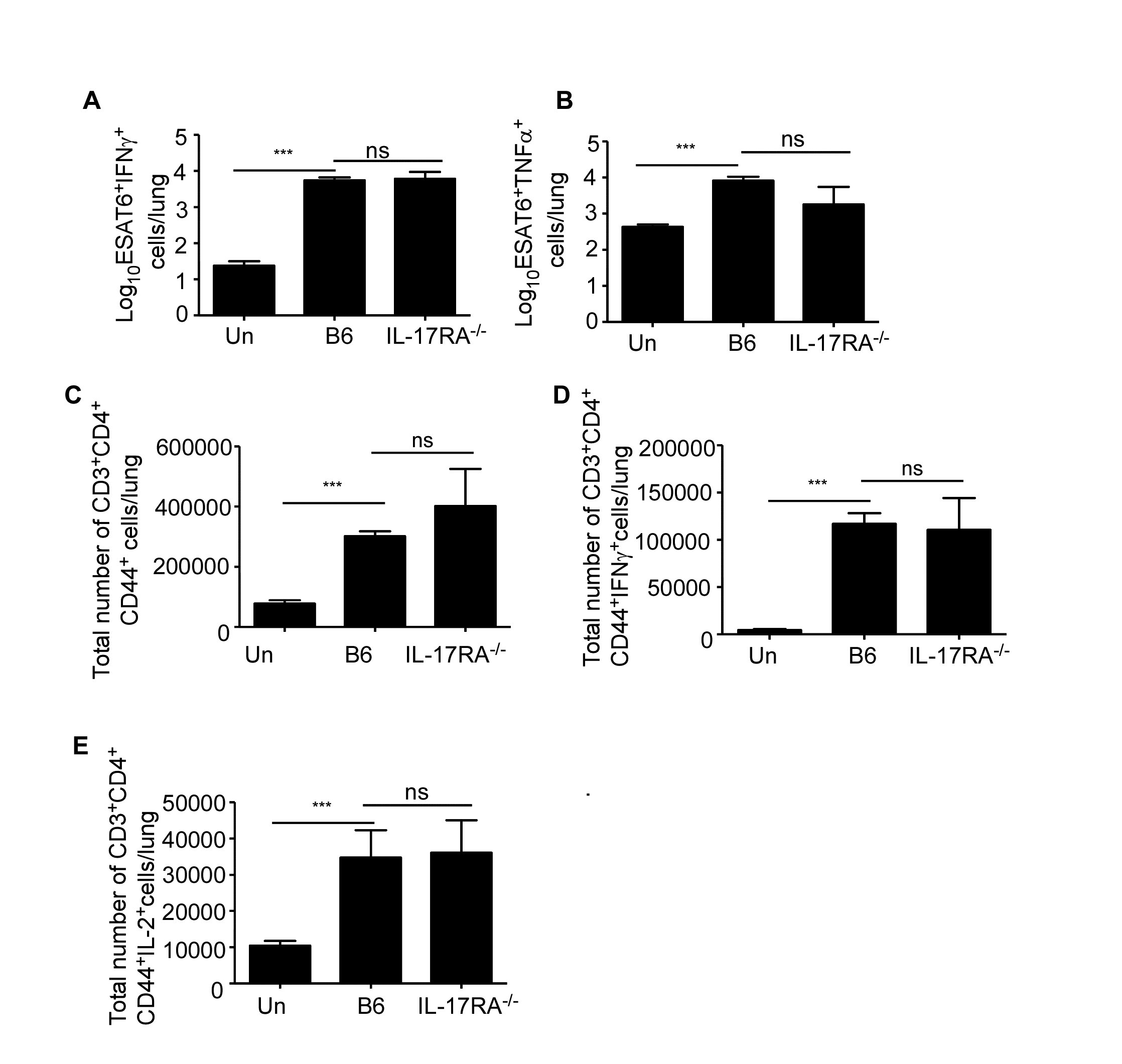

Supplement: Figure S1 — IL-17R−/− mice have unaltered Th1 responses. B6 or IL-17R−/− mice were aerosol infected with ∼100 cfu Mtb HN878 and lungs were collected on D30 post-infection. The percentage of ESAT-61–20-specific, IFN-γ (a) and TNF-α (b)-producing cells in the lungs of Mtb-infected mice and uninfected mice (Un) was determined by Mtb-specific ELISpot assay. The total number of activated lung CD4+ T cells (CD3+CD4+CD44+) (c) that produced IFN-γ (d) or IL-2 (e) was determined by flow cytometry. The data points represent values from n = 3–5 mice per group *p≤0.05, **p≤0.005, ***p≤0.0005. ns-not significant. (TIF) [file ppat.1004099.s001.tif]

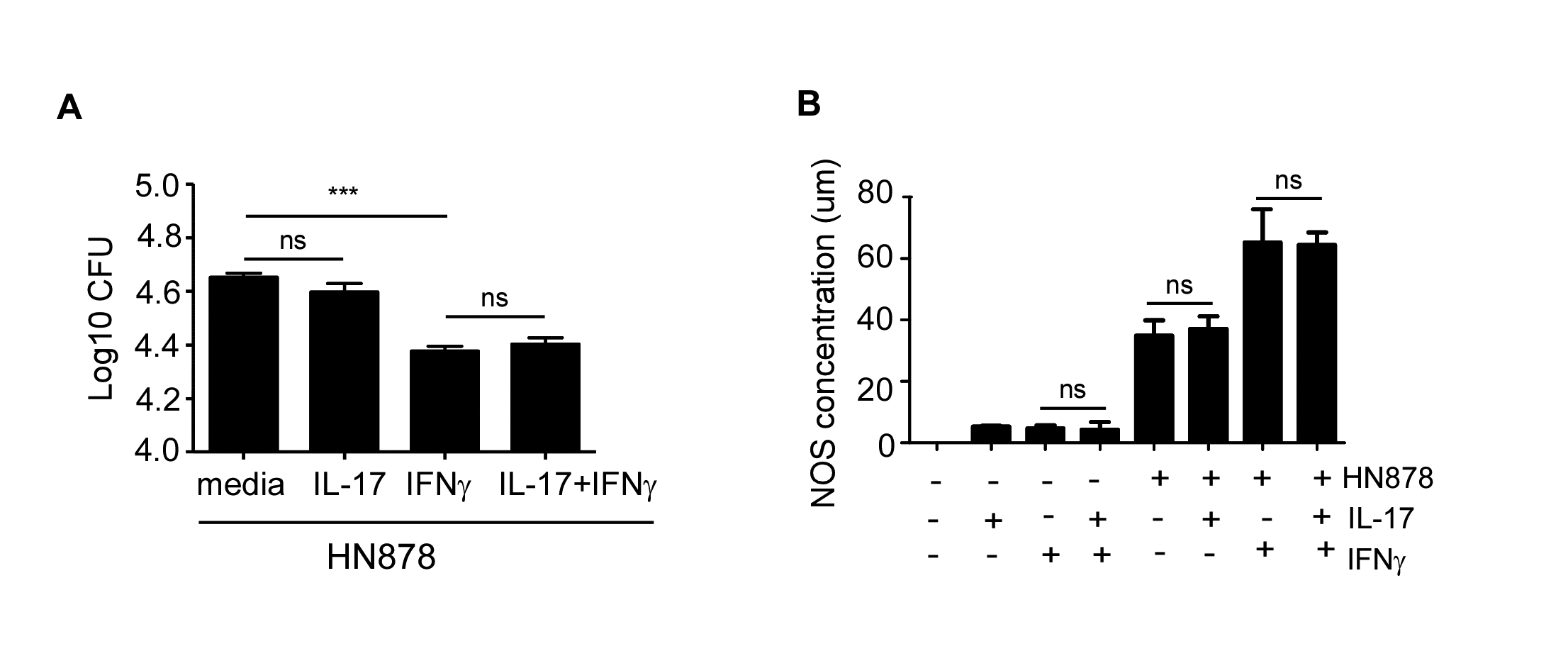

Supplement: Figure S2 — IL-17 does not directly mediate macrophage activation and Mtb control. BMDMs were in vitro infected with Mtb HN878 (MOI 5) and treated in the presence of IL-17, IFN-γ or both cytokines, and macrophage-mediated killing was determined by bacterial plating of macrophage lysates (a). Nitrite production by these macrophages in culture supernatants was determined by using Griess reaction (b). The data points represent the mean (±SD) of values from 3–5 samples. ***p≤0.0005. ns-not significant. (TIF) [file ppat.1004099.s002.tif]
